# Brain Plasticity Effects of Neuromodulation Against Multiple Sclerosis Fatigue

**DOI:** 10.3389/fneur.2015.00141

**Published:** 2015-07-03

**Authors:** Franca Tecchio, Andrea Cancelli, Carlo Cottone, Roberta Ferrucci, Maurizio Vergari, Giancarlo Zito, Patrizio Pasqualetti, Maria Maddalena Filippi, Anna Ghazaryan, Domenico Lupoi, Fenne M. Smits, Alessandro Giordani, Simone Migliore, Camillo Porcaro, Carlo Salustri, Paolo M. Rossini, Alberto Priori

**Affiliations:** ^1^Laboratory of Electrophysiology for Translational neuroScience (LET’S), Department of Neuroscience, ISTC, CNR, Fatebenefratelli Hospital – Isola Tiberina, Rome, Italy; ^2^Unit of Neuroimaging, IRCCS San Raffaele Pisana, Rome, Italy; ^3^Clinical Neurology, Catholic University, Policlinico A. Gemelli, Rome, Italy; ^4^Fondazione IRCCS Ca’ Granda, Ospedale Maggiore Policlinico and Università degli Studi di Milano, Milan, Italy; ^5^AFaR Division, Fatebenefratelli Foundation for Health Research and Education, Rome, Italy; ^6^University of Amsterdam, Amsterdam, Netherlands; ^7^University of Campus Biomedico, Psychology Service, Rome, Italy; ^8^Institute of Neuroscience, Medical School, Newcastle University, Newcastle upon Tyne, UK

**Keywords:** fatigue in multiple sclerosis, electroencephalography, transcranial magnetic stimulation, transcranial direct current stimulation, magnetic resonance imaging, electrode personalization

## Abstract

**Rationale:**

We recently reported on the efficacy of a personalized transcranial direct current stimulation (tDCS) treatment in reducing multiple sclerosis (MS) fatigue. The result supports the notion that interventions targeted at modifying abnormal excitability within the sensorimotor network could represent valid non-pharmacological treatments.

**Objective:**

The present work aimed at assessing whether the mentioned intervention also induces changes in the excitability of sensorimotor cortical areas.

**Method:**

Two separate groups of fatigued MS patients were given a 5-day tDCS treatments targeting, respectively, the whole body somatosensory areas (S1_wb_) and the hand sensorimotor areas (SM1_hand_). The study had a double blind, sham-controlled, randomized, cross-over (Real vs. Sham) design. Before and after each treatment, we measured fatigue levels (by the modified fatigue impact scale, mFIS), motor evoked potentials (MEPs) in response to transcranial magnetic stimulation and somatosensory evoked potentials (SEPs) in response to median nerve stimulation. We took MEPs and SEPs as measures of the excitability of the primary motor area (M1) and the primary somatosensory area (S1), respectively.

**Results:**

The Real S1_wb_ treatment produced a 27% reduction of the mFIS baseline level, while the SM1_hand_ treatment showed no difference between Real and Sham stimulations. M1 excitability increased on average 6% of the baseline in the S1_wb_ group and 40% in the SM1_hand_ group. Observed SEP changes were not significant and we found no association between M1 excitability changes and mFIS decrease.

**Conclusion:**

The tDCS treatment was more effective against MS fatigue when the electrode was focused on the bilateral whole body somatosensory area. Changes in S1 and M1 excitability did not correlate with symptoms amelioration.

**Significance:**

The neuromodulation treatment that proved effective against MS fatigue induced only minor variations of the motor cortex excitability, not enough to explain the beneficial effects of the intervention.

## Introduction

Fatigue is defined as “a feeling of insufficient physical and/or mental energies interfering with the usual and desired activities” (1). It is a common and highly disabling symptom in patients affected by multiple sclerosis (MS) even when other symptoms remain mild (2).

### Involvement of the motor control system in MS fatigue

To date, there is no clear evidence pointing at a single factor causing MS fatigue and fatigue complaints appear completely unrelated to both clinical variables, such as type of MS, level of disability, or disease duration, and demographic ones, such as age, gender, and education level (3). Although peripheral conditions, such as muscle weakness, may play a role, there are clear indications that much of MS fatigue has a central origin, most likely being the consequence of a failing central motor transmission to spinal alpha motor neurons (4).

### tDCS treatment targeting “whole body S1” vs. “hand SM1”

A few years ago, Cogiamanian obtained an increase of endurance against fatigue in healthy subjects by submitting them to a transcranial direct current stimulation (tDCS) (5). Recently, we applied Cogiamanian’s treatment to fatigued MS patients (6) obtaining a significant amelioration of their symptoms. In the present study, we tested two variations of Cogiamanian’s protocol on two distinct subgroups of fatigued MS patients. We submitted the first subgroup to Cogiamanian’s same treatment only replacing the original mono-hemispheric with a bihemispheric stimulation (we will call this treatment SM1_hand_).

It is known that fatigued MS patients show a much higher excitability of their primary motor area (M1) than non-fatigued patients and healthy subjects. This phenomenon has been attributed to a failure of intracortical inhibition (ICI) in frontal and M1 areas, both before and after fatiguing exercises (4). Furthermore, structural and functional data report a parietal involvement in MS fatigue symptoms (7–9), with indications of a reduced primary somatosensory area (S1) excitability (10, 11), and tDCS has been reported to enhance parieto-frontal projections (12). Also, in previous works of ours, we noticed signs of impaired communication between S1 and M1 (13).

Consequently, on the base of the above considerations, for the second subgroup, we modified Cogiamanian’s treatment to selectively direct our neuromodulation on bihemispheric whole body S1, avoiding further direct enhancement of M1 excitability (14). Cogiamanian and coworkers assessed fatigue in hand movements and stimulated the hand section of SM1 representation (5). We considered that in MS patients, the lower limbs are also primarily involved and there are no reasons to limit neuromodulation to only the section of S1 devoted to hand representation. Thus, we treated the second subgroup with a tDCS on bilateral whole body S1 (we will call this treatment S1_wb_).

### Aim

Within this theoretical frame, our present aim was to test whether a tDCS treatment, which decreases MS fatigue, induces changes in brain excitability. In particular, we intended to quantify the effects induced within M1 via transcranial magnetic stimulation (TMS)-evoked motor evoked potentials (MEPs) (15) and in S1 via median nerve (MN) evoked somatosensory evoked potentials (SEPs).

## Materials and Methods

The protocol was approved by the Ethics Committee of the “S. Giovanni Calibita” Fatebenefratelli Hospital in Rome and by Ethics Committee of Università degli Studi di Milano, Fondazione IRCCS Ospedale Maggiore Policlinico, Mangiagalli e Regina Elena.

### Study design

Both our studies (S1_wb_ and SM1_hand_ treatments) followed a double blind, sham-controlled, randomized, cross-over design (Sham/Real, Real/Sham). Patient remained blind to whether they would receive a real or a sham treatment. Patients were asked to fill out the modified fatigue impact scale (mFIS) form to score their level of fatigue. We will refer to the week before the first tDCS treatment as T0 (baseline) and to at least 4 h after the last tDCS treatment as T1. We collected electroencephalographic (EEG) and TMS sessions and mFIS scores at T0 and T1.

### Sample size estimate

We calculated the sample size using the repeatability of mFIS scores before neuromodulation treatments started. In 10 individuals with mild MS, we collected mFIS twice, 1 week apart. The average mFIS pre–post score difference was 0.1 ± 1.9, and the Intra-Class Correlation indicated a very high agreement (ICC = 0.96; *p* < 0.001). According to our previous study (6), the variability of changes after stimulation was quite larger (21.1% after real, 16.9% after sham). In order to assume the “worst” yet more realistic scenario, we did not lean on homoscedasticity and assumed both such variability values, distinguishing real and sham variances of pre–post-stimulation changes. In Tecchio et al. (6), we observed a 27% improvement after real and 7% improvement after sham treatment. To recognize as significant (alpha level = 0.05), a 20% difference between Real and Sham treatments, a sample size of 10 cases will provide a power of 90%. Notably, biomedical literature considers a 25% improvement (ere expected for Real stimulation) as a suitable threshold of clinical relevance (16) and here would correspond to a decrease of 12 mFIS points for a severely fatigued patient with 48 at baseline.

### Participants

We recruited 21 relapsing-remitting (RR) MS patients (17) experiencing fatigue [physical items mFIS score >15, Ref. (18)]. Inclusion criteria were as follows: mild physical disability [expanded disability status scale, EDDS (19) cut-off score of ≤3], absence of depression (no pharmacological treatment), absence of clinical relapse, or radiological evidence of disease activity over the last 3 months. Exclusion criteria were as follows: use of symptomatic drugs, which may affect the level of fatigue, depression, and anxiety within the past 3 months (20), epilepsy or other central/peripheral nervous system comorbidities and any systemic conditions, which may cause fatigue (e.g., anemia and pregnancy). All patients underwent brain magnetic resonance imaging (MRI) for exclusion criteria assessment. In addition, a detailed clinical history was collected including active disease modifying therapy (DMT), disease duration, annual relapse rate, and depression level (Beck depression inventory, BDI). Fine hand motor control was evaluated by nine hole peg test (9HPT) scores collected separately for left and right sides.

### MRI exam and measure estimate

#### Image Acquisition

In each patient undergoing S1_wb_ treatment, brain imaging was performed by an Achieva 1.5-T scanner (Philips Medical Systems, Best, The Netherlands), with 33 mT/m gradients, online 2D/3D geometric distortion correction, and an 8-channels head Phased-Array coil with parallel imaging capabilities (SENSE). All sequences were acquired with contiguous slices and full brain coverage.

Exclusion criteria (no active lesions) were assessed based on T1-Spin Echo images before and 5 min after intravenous injection of a contrast agent. Lesions estimates were based on T2 Dual Echo images (see: column 2 of Attachment 2) and 3D-FLAIR (see: column 4 of Attachment 2).

T1-3D Fast Field Echo sequences with full brain coverage (MPRAGE, TR/TE/FA = 8.6 ms/4 ms/8°; 170 contiguous sagittal slices 1.2 mm thick without gap, mtx1922) were used for the 3D reconstruction of the brain structure in order to personalize the tDCS electrode.

#### Image Post-Processing Computations

##### Lesion load

A semi-automated region of interest (ROI) approach was used to trace hyperintense lesions in the white matter (WM) on T2-weighted images, following strategies previously described [Ref. (11); Jim 5.0, Xinapse Systems Ltd., Leicester, UK, Attachment 3]. ROIs were identified by consensus of two investigators (Giancarlo Zito and D. Lupoi) blind to patients’ clinical data. The total lesion volume (TLV) was computed. Lesion relative fraction (LrF) was computed as the ratio of the TLV over the WM volume in order to normalize for inter-subject head volume variability.

### Whole body S1 personalized electrode shaping and positioning

#### Personalized Electrode Shaping

**Figure 1:**
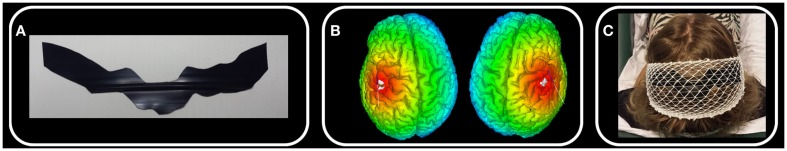
**Whole body S1 personalized electrode**. In one exemplificative subject, we schematize the main steps of electrode personalization [Ref. (14), see Materials and Methods). **(A)** After drawing the left and right central sulci using SoftTaxic software from individual 3D MRI, we fit this line by 2 cm wide parallelograms and we cut the electrode from a conductive silicon band. **(B)** We position the personalized stimulating electrode by proper neuronavigation procedure along the central sulcus with the center of the electrode crossing the nasion-inion line. **(C)** S1_wb_ personalized electrode and the cathode electrode positioned on Oz.

A few days before the experimental session, each subject underwent a structural brain MRI exam with a 1.5-T scanner (Achieva, Philips Medical Systems, Best, Netherlands; MPRAGE contiguous sagittal slices with full brain coverage). MRI data were elaborated with SofTaxic Neuronavigation System ver. 2.0 (www.softaxic.com, E.M.S., Bologna, Italy), which delivered the volumetric reconstruction of the individual brains and the cortical folders. The stereotaxic procedure for the personalization of each electrode included the following steps [Figure 1; (14)]: (1) the line of the central sulcus shown by the navigator is manually transferred onto a paper sheet firmly fixed onto the patient’s scalp; (2) on paper, 2 cm-long segments are drawn perpendicularly from a number of equidistant points of the central sulcus line in the anterior direction. The number of equidistant points is chosen to obtain a total electrode surface of 35 cm^2^, which the literature widely reports as the recommended size for a direct current intensity of 1.5 mA. (3) The shape obtained on paper is transferred onto a commercial band of conductive silicone. The latter is 0.2 mm thick and has a 1 mm diameter channel running along its length. The electrode is manually cut along the contour, making sure that the channel remains roughly at the center of the band’s length. (4) A standard electric wire, which will deliver the 1.5 mA direct current, is finally placed inside the channel.

Following the SofTaxic navigator, the electrode was positioned 1.5 cm posterior and 0.5 cm anterior to the central sulcus, centered on the nasion-inion line. Cathode electrode (6 cm × 14 cm) was positioned on Oz. Contact with the subject’s head was facilitated by a conductive gel and an elastic cotton net maintained the electrodes stable along the entire session (Figure 1).

### Transcranial direct current stimulation (5-day treatment)

Transcranial direct current stimulation was delivered by an electrical stimulator through a constant current unit and an isolation unit [SM1 (21); S1-Eldith Stimulator by NeuroConn, Ilmenau, Germany]. Anode electrode was positioned as described above. Cathode electrode was under the chin for the SM1 stimulation and on Oz for the S1 stimulation.

The 1.5-mA constant current was applied for 15 min once a day for five consecutive days, according to previous studies against pain (22, 23). In particular, a 1.5-mA current strength produces a current density of about 0.04 mA/cm^2^ for the anode electrode of 35 cm^2^ (5, 24), which is well below safety thresholds. Cathode electrode size was of 84 cm^2^, resulting in a current density of 0.02 mA/cm^2^ under this electrode, corresponding to a non-effect current density in this reference region (25, 26). Impedances were below 10 kΩ throughout the stimulations. Sham condition consisted of 4 s of active stimulation at the beginning and at the end of each day’s 15-min stimulation. At debriefing, no subject reported to feel any difference across tCSs.

### Transcranial magnetic stimulation to probe cortical excitability changes in M1

**Figure 2:**
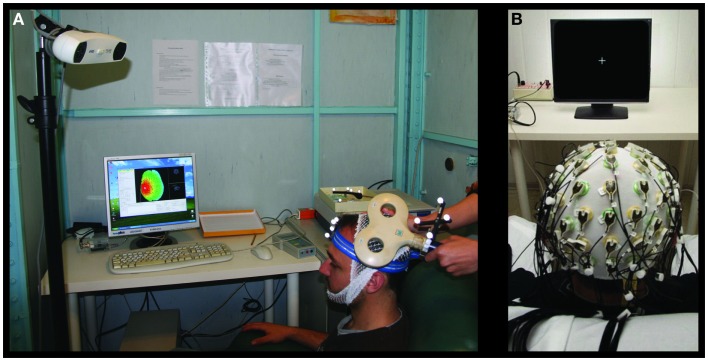
**Transcranial magnetic stimulation and EEG settings for brain plasticity assessment**. Experimental settings for the MEP **(A)** and SEP **(B)** recordings.

Single-pulse TMS was performed through a standard focal coil (diameter of each wing 70 mm) connected with a Bistim 200 module (The Magstim Company Ltd., Whitland, UK). We recorded TMS MEPs from left and right *opponens pollicis* (OP) by surface electrodes in a belly tendon montage (2.5 cm apart). Following international standards, we identified the “hot-spot” of the right OP muscle and the corresponding resting motor threshold (RMT) (27, 28). Thereafter, we maintained the coil position – digitized and monitored throughout the whole session by the SofTaxic neuronavigator – by means of a support arm (Figure 2A).

Transcranial magnetic stimulation intensity was settled at 120% RMT and 20 MEPs were then collected in complete relaxation while TMS was delivered with an inter-stimulus interval randomly ranging between 5 and 7 s. The whole procedure was repeated in the other hemisphere to obtain left OP motor cortical representation.

### Electroencephalographic study to probe cortical excitability changes in S1

#### Electrophysiological Data Recording

Electroencephalographic signals were recorded with a 64-channel actiChamp System (Brain Products GmbH, Gilching, Germany, Figure 2B). The montage included Fz derivation for reference and FPz for ground. EEG signals were sampled at 5 kHz and a preconditioning 0.1–1500 Hz bandpass filtering was applied.

#### Median Nerve Stimulation

All subjects sat comfortably on an armchair during the experiment. In order to induce somatosensory evoked responses following a painless thumb twitch, their MN was stimulated at the wrist with a constant current electrical stimulator (Model DS3, Digitimer Ltd., Hertfordshire, UK), using standard parameters (cathode proximal, 250 ms inter-stimulus interval, 0.2 ms duration, above motor threshold intensity).

Left and right MNs were separately stimulated for 5.5 min, totaling about 1300 artifact-free trials, which were stored for off-line analysis. The SEP epochs ranged from 10 ms pre to 100 ms post-stimulus. Epochs whose voltage amplitudes exceeded ±100 μV at the EOG electrode as well as those containing saturating artifacts were rejected.

All amplitude values referred to the 5–10 ms post-stimulus interval. The amplitude of the N20 component was measured as the first negative peak between 18 and 23 ms. The N20–P25 complex was determined as the difference between the N20 peak and the subsequent positivity peak (P25), occurring at a latency of around 23–29 ms.

For purposes of the present study, we used the typical bipolar derivation used to assess SEPs [C3–C4, Ref. (29)].

### Statistical analysis

After checking the distribution of MEP and SEP amplitudes (as tested by Shapiro–Wilk test), we applied, when necessary, suitable transformations in order to achieve a better approximation to gaussianity and a good control of outliers.

To test the effects of the 5-day tDCS on MFIS, MEP, and SEP variables, analyses of variance (ANOVA) for repeated measures were performed with *Stimulation* (Real, Sham) and *Treatment* (pre-, post-tDCS treatment) as within-subjects factors. Within-subjects factor *Hemisphere* (Left, Right) was included for MEP and SEP, which had been collected bilaterally since we performed a bilateral stimulation. A similar approach was used for the effects on fine hand motor control measure, with the 9HPT submitted to the ANOVA with *tDCS Intervention* (Pre, Post), *Stimulation* (Real, Sham), and *Hand* (Right, Left) within-subjects factors. We performed separate ANOVA designs in the two patients’ subgroups stimulated on bilateral S1_wb_ or SM1_hand_. Significance threshold was set to 0.050 and we reported trends for *p* < 0.100.

## Results

The 21 patient cohorts presented a mild clinical picture in accordance to the inclusion criteria (Table 1). The two electrode-dependent subgroups displayed homogenous clinical features (Table 1).

**Table 1:** **Demographic and clinical profile of people with MS**.

	Sex	Age	Dis Dur	EDSS	BDI	mFIS	LrF	9HPT
S1_wb_	9F/4M	45.8	7.6	*1.5*	12.7	41.6^a^	0.38	20.8
		(7.6)	(8.2)	[0–3.5]	(3.5)	(7.5)^a^	(0.48)	(4.9)
SM1_hand_^b^	6F/2M	38.1	13.5	*2*	11.0	57.1		
		(9.8)	(4.2)	[1–2.5]	(5.1)	(19.9)		
	*p*	*0.080*	*0.068*	*0.254*	*0.438*	*0.062*		

*M, male; F, female; Mean or Median in italics and SD, standard deviations () or ranges [min, max] across the group of: Dis Dur, disease duration; Scores of: EDSS, expanded disability status scale; BDI, Beck depression inventory; LrF, lesion relative factor; MFIS, modified fatigue impact scale, and 9HPT, time (s) to execute right hand 9-Hole Peg Test at baseline*.

*^a^mFIS 1-week apart repetition was 41.5, SD 6.1 (see Study Design). MRI-derived measures (LrF) and 9HPT were not collected in the SM1_hand_ group*.

*^b^Two out of the 10 patients of the SM1_hand_ group dropped out. In the last row, the significance og the comparison between the two groups*.

### Fatigue levels

#### Whole Body S1 Stimulation (S1_wb_)

Analyses of variance indicated that mFIS changes were related to the type of stimulation (Real or Sham) when the bilateral personalized S1_wb_ electrode was used [*Stimulation* × *Treatment* interaction *F*(1,8) = 9.692, *p* = 0.014, (6), Table 2]. Fatigue resulted reduced after real stimulation (*post hoc* comparison *p* = 0.002, 31.0 ± 12.0 post- vs. 42.1 ± 7.9 pre-stimulation), whereas there were no changes after the sham stimulation [*post hoc* comparison *p* = 0.901, 34.8 ± 10.4 post- vs. 37.2 ± 7.0 pre-stimulation, (6), Table 2]. After real stimulation, the mean fatigue reduction was 28% of the baseline (range between 2 and 76%), and 8% after sham (range between −11 and 38%, paired-samples *t*-test real vs. sham, *p* = 0.016).

**Table 2:** **Transcranial direct current stimulation treatment effects on fatigue**.

	Real		Sham		
	T0	T1	T0	T1	*p*
S1_wb_	**42.1**	**31.0**	37.2	34.7	0.014
	(7.9)	(12.0)	(7.0)	(10.4)	
SM1_hand_	57.8	42.1	55.5	52.1	0.239
	(19.9)	(17.2)	(26.6)	(22.0)	

*Mean and SD of fatigue scale (mFIS) across patients before and after 5-day tDCS treatment, stimulating bilateral either whole body S1 (S1_wb_) or hand section of SM1 (SM1_hand_). p is the significance of the Stimulation × Treatment interaction effect. In bold, values with significant difference between pre- and post-treatment, as estimated by post-hoc comparison whenever the Stimulation × Treatment interaction effect was significant*.

### tDCS treatment effect on fine hand motor control (9HPT)

In the S1_wb_ group, 9HPT of the right hand correlated with both EDSS and physical items of MFIS (Pearson’s *r* = 0.736, *p* = 0.015 and *r* = 0.744, *p* = 0.014, respectively). It should be noted that the correlation between MFIS_phys and 9HPT remains substantially stable after correction for EDSS (partial correlation *r* = 0.602, *p* = 0.086). The lesion load was not associated with any clinical or fatigue-related measure (LrF with EDSS, BDI, total or physical MFIS *p* > 0.200 consistently).

The full model ANOVA evidenced, in addition to the right hand performing better than the left [*Hand* factor *F*(1,8) = 5.749, *p* = 0.043, overall average 20.3 ± 4.6 and 22.6 ± 4.3 s, respectively], that the two hands’ performances were differently affected by the intervention [*Hand* (Right, Left) × *tDCS intervention* (Pre, Post) × *Stimulation*(Real, Sham) effect *F*(1,8) = 5.697, *p* = 0.044]. Repeating the reduced models for each hand separately, we observed that the left hand did not change after the 5-day stimulation, while the right hand 9HPT changed in terms of dependence on whether the stimulation was real or sham [*Stimulation* × *tDCS intervention* effect *F*(1,8) = 5.680, *p* = 0.044]. The *post hoc* comparison showed that, after the real stimulation, the time required to execute the 9HPT decreased (two-tails paired *t*-test *p* = 0.038, with average 21.1 ± 4.9 pre and 19.8 ± 3.8 s post values], while it was unchanged by sham stimulation (*t*-test *p* = 0.401). No association emerged among post-tDCS values of MFIS regarding either total or physical and 9HPT scores.

#### Hand SM1 Stimulation (SM1_hand_)

No interaction *Stimulation* × *Treatment* effect was observed when SM1_hand_ electrode was used (*p* > 0.200, Table 2), indicating that effects of real and sham stimulations on fatigue levels were not clearly different.

### M1 excitability

No differences were observed in RMTs, stimulation intensities or MEP latencies when compared between hemispheres, between stimulation types (Real or Sham) or treatments (pre–post-stimulation) (*p* > 0.200 consistently). In the S1_wb_ group, the mean of RMT across all conditions was 58.4 ± 2.6% of the maximal stimulator output, TMS intensity was 70.2 ± 3.1%, MEP latency was 27.3 ± 3.2 ms. In the SM1_hand_ group, the RMT decreased after stimulation (paired-samples *t*-test *p* = 0.004; pre 59.2 ± 18.8 and post 55.0 ± 17.9% of the maximal stimulator output), while the latency did not change 24.4 ± 2.6 ms. MEP latency was associated to the MS severity (EDSS–MEP latency Pearson’s coefficient *r* = 0.880, *p* = 0.021).

Motor evoked potential amplitude distribution definitely differed from a Gaussian and we obtained a good fit by natural logarithmic transformation (Shapiro–Wilk *p* > 0.200 consistently).

No association was evident between the order of MEP collection and its amplitude (Pearson’s correlation *p* = 0.607). Mean MEP amplitude, estimated as exponential back-transformation of mean of logarithm-transformed MEP amplitudes, were 171.5 ± 1.8 for the right OP and 145.3 ± 1.9 for the left OP. No difference of baseline MEP amplitudes was observed between Real and Sham stimulations (*t*-test *p* = 0.380 averaging right and left values).

In the S1_wb_ group, the ANOVA on the MEP amplitude showed a trend interaction effect *Stimulation* × *Treatment* (*p* = 0.073), which corresponded to an increase of MEP amplitude after the real stimulation (*Treatment* effect, *p* = 0.037), absent after Sham (*p* = 0.275). The average increase with respect to the baseline level was 6.0% ranging between 0.2 and 22.6% of baseline level (MEPpost–MEPpre/MEPpre of logarithm-transformed MEP amplitudes averages).

In the SM1_hand_ group, MEP amplitude increased after the real stimulation (*Treatment* effect, *p* = 0.021). The average increase with respect to the baseline level was 40.4% ranging between 16.7 and 76.0% of baseline level.

No association emerged among post-tDCS MEP values and 9HPT scores.

### S1 excitability

No effects were observed in the N20 SEP component between the two hemispheres or the two stimulations (pre–post-stimulation, Real or Sham, *p* > 0.200 consistently). The N20–P25 complex showed a tendency to increasing after the stimulation, but no *Stimulation* × *Treatment* effect was found (*p* > 0.200).

### Relationships between S1 and M1 excitability and MFIS variations

Fatigue level changes did not correlate with variations in M1 excitability in either of the S1_wb_ or SM1_hand_ subgroups (*p* > 0.200 in both cases).

## Discussion

Our 5-day tDCS stimulation targeting the bihemispheric whole body somatosensory region significantly decreased MS fatigue. In addition, hand muscle MEPs showed that that stimulation modified M1 excitability, whereas MN SEPs showed no evidence of changes in S1 excitability.

### Mechanisms behind regional dependence of tDCS treatment efficacy (i.e., S1-whole body vs. SM1-hand)

Overall, the 5-day tDCS treatment targeting the bilateral S1_wb_ representation showed the *Stimulation* × *Treatment* effect, which was lacking in the SM1_hand_ intervention. Noteworthy, the average decrease in mFIS score was 15.6 after real SM1_hand_ stimulation – larger than the 11.1 point decrease observed after real S1_wb_ stimulation, with similar baseline levels – and the specific paired *t*-test comparison was significant (*p* = 0.030). We also analyzed why an average net (real minus sham) SM1 effect of 15.6–3.4 = 12.2 was not significant while an average net S1 effect of 11.1–2.4 = 8.7 was significant, with comparable SD (a little bit larger in S1, indeed) (although such a comparison is irrelevant in the absence of a significant interaction). The reason lies in the different correlation patterns: within the SM1_hand_ subgroup, correlations are absent between Sham and Real baseline levels, as well as between fatigue scale values from other time points, contrasting clear (and expected) correlations in the S1_wb_ subgroup (Table 3).

**Table 3:** **Intra-cohort fatigue levels correlation**.

	S1_wb_		SM1_hand_	
	**ρ**	*p*	**ρ**	*p*
Real vs. Sham T0	**0.718**	0.045	−0.299	0.471
Real T0 vs. T1	**0.840**	0.002	0.403	0.323
Sham T0 vs. T1	**0.957**	0.000	−0.054	0.900

*Pearson’s correlation coefficients (ρ) and significance (*p*) between real and sham baseline mFIS scores (T0) and between mFIS scores at baseline (T0) and after-treatment (T1) both after real (second row) and sham (third row) treatments, for both the SM1_hand_ and S1_wb_ subgroups. In bold, significant correlations*.

The S1_wb_ treatment was more effective than over SM1_hand_ (21) and than over the left prefrontal cortex (30). This comparative result strengthens the working hypothesis, which guided the development of the S1-whole body personalized electrode. In fact, data available in the literature document a failure of the inhibitory mechanisms in the frontal and primary motor (M1) areas involved in motor planning (4), a reduced M1 ICI before and after fatiguing exercises (4), and an increase in M1 excitability (4) in fatigued vs. non-fatigued MS patients and to healthy subjects. Concurrently, together with excessive excitability of M1, we observed signs of a reduced S1 excitability (10). Moreover, we observed an altered parieto-frontal projection, mainly involving S1 and M1, in fatigued vs. non-fatigued MS patients (13, 31). Thus, we decided to neuromodulate to enhance selectively the excitability of S1, avoiding a direct enhancement of M1 excitability (as occurs with SM1 electrode), to further support the parieto-frontal projection already observed by tDCS (12).

### Suitability of differentiated effects targeting S1 vs. SM1

Transcranial direct current stimulation-generated modulations of cortical excitability can be focused by means of proper sizing and positioning of the stimulation electrode. Since tDCS efficacy is determined by the current density (i.e., current strength/electrode area), we can obtain increased focality by reducing the electrode size while keeping a constant current strength. In the motor system, Nitsche and colleagues (32) compared tDCS effects on central representations of two muscles, first dorsal interosseus (FDI) and abductor digiti minimi (ADM), by measuring MEPs. Stimulation with small electrodes (3.5 cm^2^) generated focal effects, with different MEP amplitude increases for the two muscles (32). The protocol we are proposing actually requires less focality than Nitsche’s, where a discrimination of M1 neuronal pools controlling the two hand muscles was sought. In fact, we intend to stimulate motor vs. sensory regions. However, while positioning of tDCS electrodes in M1 stimulation can be guided by TMS, which induces responses from specific muscles, a neuronavigation system is required when stimulating S1 vs. M1 to precisely identify the central sulcus. Modern frameless stereotaxic systems allow navigation on the subject’s structural MRI-derived brain representation, providing high-spatial precision with accuracy in the range of millimeters (33). In our experimental setup, we used precise topographical determination of the central sulcus in placing the S1_wb_ electrode (6, 14, 25, 34).

### tDCS targeting bilateral vs. mono-hemispheric regions

Multiple sclerosis fatigue is not associated to mono-hemispheric prevalence, as shown by electrophysiological (10) and neuroimaging data (9). Thus, via the tDCS intervention, we targeted bilateral (35) either whole body S1 or hand SM1. In the present results, we observed bilateral M1 enhancement, documenting that bilateral stimulations of a homologous area do not cancel out. This hypothesis of ineffective bilateral M1 stimulation is derived from the well-known motor system organization, with M1 of one hemisphere inhibiting M1 of the other hemisphere. Through bilateral stimulation of homologous M1 areas, the concurrent increase of inhibition induced by the increase in excitability of one hemisphere might thus cancel out the increase in excitability in the other hemisphere. However, we can reject such a hypothesis, and we can also speculate that a relevant component of the presently observed neuromodulation operates directly on local pyramidal neurons, and not via inhibitory or excitatory networks beneath the electrode (36, 37).

### Brain plasticity induced by S1 stimulation

We did not find evidence of S1 excitability changes induced by S1_wb_ tDCS treatment, as measured by the typical SEP assessment. This can be due to two causes. The first is that the SEP gives an indirect assessment of cortical pyramidal neurons with respect to TMS-derived MEP. In fact, TMS stimulates pyramidal neurons and the MEP muscle response gives a measure of cortical excitability with as a single-station-pathway (only the spinal cord relay in between). Instead, the pathway between MN stimulation and S1 (here assessed by single-derivation SEP) includes spinal cord, brain stem, and thalamic relays. The second reason can be poor sensitivity of EEG-derived SEP analysis. In addition, we found more effects in M1 than in S1, which can be due to non-selective S1 stimulation. Via simulations (in progress), we are in fact observing that the induced current density is slightly prevalent in S1 but it is of a comparable intensity also in M1.

### Study limitations

We did not study the two datasets (S1_wb_ and SM1_hand_ 5-day tDCS treatments) in a single statistical model, since a different anode electrode size (anode electrode area of 70 cm^2^ for SM1 and 35^2^ for S1) and a different reference position (on Oz or on the left shoulder) were used.

Here, we investigated somatosensory evoked responses, since we performed a stimulation planned to focus on S1. We started from a standard single derivation in each hemisphere to assess SEP changes. Nevertheless, we collected 64-channel EEG data to further investigate cortical effects. In particular, source analysis will allow future investigations of our main hypothesis of a modification induced by the tDCS treatment on sensory-motor functional connectivity.

## Conflict of Interest Statement

The authors declare that the research was conducted in the absence of any commercial or financial relationships that could be construed as a potential conflict of interest.
